# APE1 overexpression is associated with poor survival in patients with solid tumors: a meta-analysis

**DOI:** 10.18632/oncotarget.19814

**Published:** 2017-08-02

**Authors:** Chun-Ling Yuan, Fan He, Jia-Zhou Ye, Hui-Ni Wu, Jin-Yan Zhang, Zhi-Hui Liu, Yong-Qiang Li, Xiao-Ling Luo, Yan Lin, Rong Liang

**Affiliations:** ^1^ First Department of Chemotherapy, Affiliated Tumor Hospital of Guangxi Medical University, Nanning, 530021, Guangxi Zhuang Autonomous Region, P. R. China; ^2^ College of Arts and Sciences, University of South Florida, Tampa, FL, 33620, USA; ^3^ Department of Hepatobiliary Surgery, Affiliated Tumor Hospital of Guangxi Medical University, Nanning, 530021, Guangxi Zhuang Autonomous Region, P. R. China; ^4^ School of Public Health, Sun Yat-sen University, Guangzhou, 510080, Guangdong, P. R. China

**Keywords:** APE1, IHC, meta-analysis, prognosis

## Abstract

APE1 is known as a key mediator of DNA damage repair pathways, and its clinical significance in different types of cancer is well studied. Herein, we performed a meta-analysis to determine the association of APE1 expression and survival in different types of solid cancer. We searched all eligible publications in PubMed, Web of Science and Embase platforms from inception to January 2017 and found 15 relevant manuscripts. Overall survival (OS), 12- and 36-month survival rates, and hazard ratios (HRs) were extracted and analyzed. Heterogeneity and publication bias were also assessed. A subgroup analysis of the different subcellular locations of APE1 was also conducted. Patients with higher APE1 levels demonstrated lower 12- and 36-month survival rates than those with low APE1 levels (HR 2.00, 95% CI 1.33–3.00, *P* = 0.0009; HR 1.84, 95% CI 1.19–2.84, *P* = 0.006). Importantly, the pooled analysis showed that high levels of APE1 predict shorter OS (HR 1.44, 95% CI 1.13–1.83, *P* = 0.003). Subgroup analysis revealed that both nuclear and cytoplasmic expression levels of APE1 are important indicators of poor prognosis in solid tumors.

## INTRODUCTION

Apurinic/apyrimidinic endonuclease/Ref-1 (APE1), the mammalian ortholog of *Escherichia coli* Xth, is a multifunctional protein with both DNA base excision repair (BER) and cell oxidative stress response capabilities and plays a key role in the incision of AP sites and the generation of 3’OH in basic reactions of the BER pathway [1]. Reflecting its other name of redox effector factor 1 (Ref-1), it reacts to different cellular stress by regulating the activity of various transcription factors, such as early growth response protein-1 (Egr-1), nuclear factor-κB (NF-κB), p53, hypoxia inducible factor-1α (HIF-1a) and activator protein-1 (AP-1) [2], which are involved in various cellular processes, including survival, cell cycle, cell growth, stem cell properties and inflammatory pathways. The main mechanism by which APE1/Ref-1 controls transcriptional factor activities is through reducing cysteine residues, thereby activating their DNA-binding activity [3, 4]. Thus, APE1 is thought to be the hub protein of cellular regulation.

Unsurprisingly, APE1 has been found to be involved in various human diseases including cancer, cardiovascular disease, and neurodegenerative diseases. Up-regulated or activated APE1 has been identified in several tumors, such as non-small cell lung cancer (NSCLC), gastric cancer, esophageal squamous cell carcinoma (ESCC) and bladder cancers [5–8], and is associated with a worse prognosis. APE1 levels are also reported to be a predictive marker for sensitivity to chemotherapy in NSCLC patients [9]. Moreover, the inhibition of APE1 can significantly enhance the anti-tumor effects of chemotherapy in cancers [10]. Thus, APE1 appears to be a very promising therapeutic target for cancer patients.

However, in some types of cancer, including hepatocellular carcinoma (HCC) and breast cancer, it has the opposite effect [11–13], and high levels of APE1 are favorable for the prognosis of patients. Some contradictory results are reported in glioma, NSCLC and gastric tumors, showing the complex role of APE1 in tumors [14]. What is more, cytoplasmic and mitochondrial localization of APE1 has been observed independently by several groups, which is unexpected for APE1, presumably a nuclear protein [15]. To the best of our knowledge, APE1 is not activated in the cytoplasm. Thus, it is necessary to investigate the prognostic value of APE1 overexpression in different solid tumors.

In this study, we found fifteen qualifying studies of the prognostic role of APE1 in eight types of tumors. A systematic meta-analysis was conducted to evaluate the prognostic role of APE1 in solid tumors, providing a novel reference for further therapeutic studies of APE1.

## RESULTS

### Study characteristics

**Figure 1:**
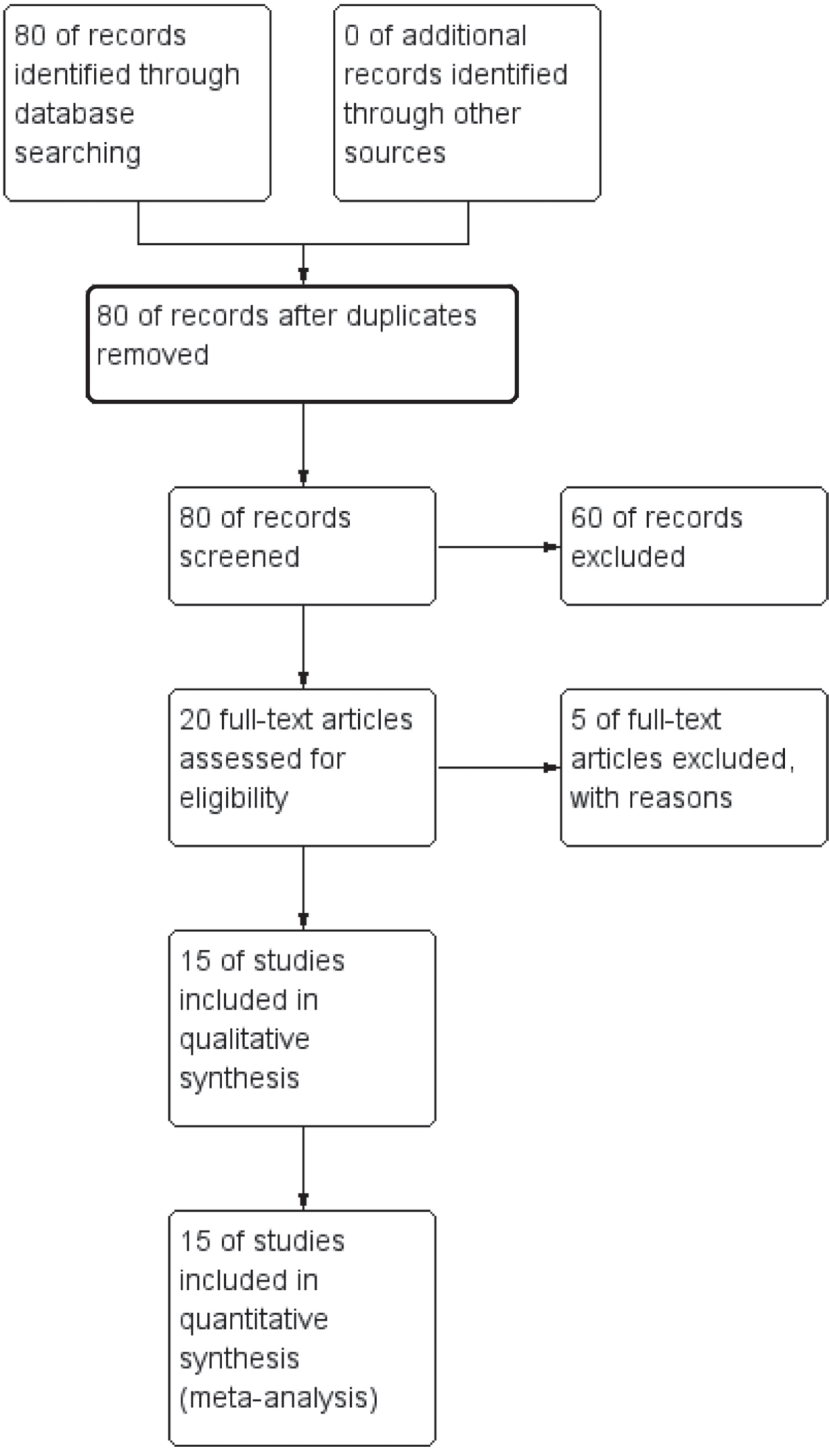
Flow diagram of the literature search strategy in the meta-analysis

A total of 80 potentially relevant publications were retrieved after the initial database searches, and 15 eligible studies were finally included in the study, comprising 1574 patients for analysis. A flow diagram of the study selection process is presented in Figure 1. The main characteristics of the 15 studies are reported in Table 1. The studies were conducted in 4 countries (China, Korea, Italy and the UK) and published from 2007 to 2016. Cancer types of the patients included lung carcinoma, ESCC, gastric carcinoma, ovarian carcinoma, HCC, locally advanced rectal cancer, breast cancer, glioma and osteosarcoma [6, 9–14, 16–23]. APE1 expression was evaluated by IHC in all 15 studies in our meta-analysis. All studies examined the association between APE1 positive expression and OS. Among them, seven studies reported that APE1 expression was detected in both the nucleus and cytoplasm, while five studies found only nuclear expression, and three studies found only cytoplasm expression. High expression of APE1 predicts a worse prognosis in 12 studies, while a better prognosis is observed in 3 studies. These studies obtained quality assessment scores ranging from 5 to 8.

**Table 1:** Main characteristic and results of the 15 included studies

Study	Year	Tumor type	Patient source	PN	Stages	Methods	HIGH	Low	Subcellular location (N/C)	Median follow up (Months)	Out-come	12-month-SR(H)	12-month-SR(L)	36-month-SR(H)	36-month-SR(L)	HR	95% High	95% Low	Result (F or U)	QA
Zhang	2016	Lung	China	172	III-IV	IHC	126	46	N+C	11(1-43)	OS	48.66	70.47	9.06	9.06	1.19	1.74	0.81	U	8
Wei	2016	Lung	China	78	II-IV	IHC	56	22	N+C	NA	OS	57.75	76.96	12.29	15.27	1.31	2.34	0.73	U	7
Wu	2010	Lung	China(Taiwan)	100	I-III	IHC	49	51	C	36.1(3.3–68.9)	OS	NA	NA	96.72	96.7	2.243	3.855	1.305	U	7
Woo	2014	Breast	Korea	239	I-III	IHC	177	62	N	61	OS	98	100	27.92	67.36	0.78	2.39	0.25	NA	8
Wang	2009	Lung	China	103	I-III	IHC	76	27	N+C	NA	OS	94.75	99	NA	NA	2.47	6.02	1.01	U	6
Han	2014	ESCC	China	93	I-IV	IHC	69	24	N	35(0.13-52)	OS	NA	NA	26.56	47.99	1.328	2.777	0.635	NA	7
Wei	2016	Gastric	China	65	I-IV	IHC	50	15	N	28.5	OS	90.2	99.8	32.95	50.51	2.57	6.18	1.07	U	7
Qing	2015	Gastric	China	107	NA	IHC	93	14	N+C	42	OS	84.01	92.53	43.2967	28.5714	1.42	3.24	0.62	U	7
Perry	2014	Glioma	UK	60	NA	IHC	35	25	N	12.8(0.23-111.7)	OS	78.02	62.6	9.39	35.79	0.53	1.02	0.28	F	6
Ren	2014	Osteo- sarcoma	China	80	I-III (Enneking staging)	IHC	55	25	N+C	NA	OS	61.17	83.69	76.7176	92.7439	1.77	2.9	1.07	U	7
Kim	2012	LARC	Korea	83	I-III	IHC	32	51	C	82(18-114)	OS	100	100	57.5713	62.8788	2.05	4.67	0.9	NA	7
Londero	2014	Ovarian cancer	Italy	73	I-IV(FIGO)	IHC	37	36	N	NA	OS	85.61	93.94	NA	NA	1.74	3.41	0.88	U	7
Maso	2007	Hepatocellular carcinoma	Italy	47	G1-G4	IHC	NA	NA	C	NA	OS	NA	NA	NA	NA	2.2	4.3	1.1	U	5
Kan	2015	Hepatocellular carcinoma	China	128	I-IV	IHC	86	42	N+C	10	OS	NA	NA	68.35	78.25	0.771	1.13	0.526	NA	6
Hsia	2016	Oral squamous cell carcinoma	China(Taiwan)	146	I-IV	IHC	77	69	N	NA	OS	83.44	91.96	9.06	9.06	1.825	3.287	1.014	U	7

NA: Not applicable

LARC: Locally advanced rectal cancer

PN: patient number

Subcellular location (N/C): N: nucleus; C: Cytoplasm

M/U M: Multivariate; U: Univariate

Result: F: Favorable; U: Unfavorable

QA: quality assessment

12/36-month-SR(H): 12/36-month survival rats (High APE1 level); 12/36-month-SR(L): 12/36-month survival rates (Low APE1 level) HR: hazard ratio

### Meta-analysis results

**Figure 2:**
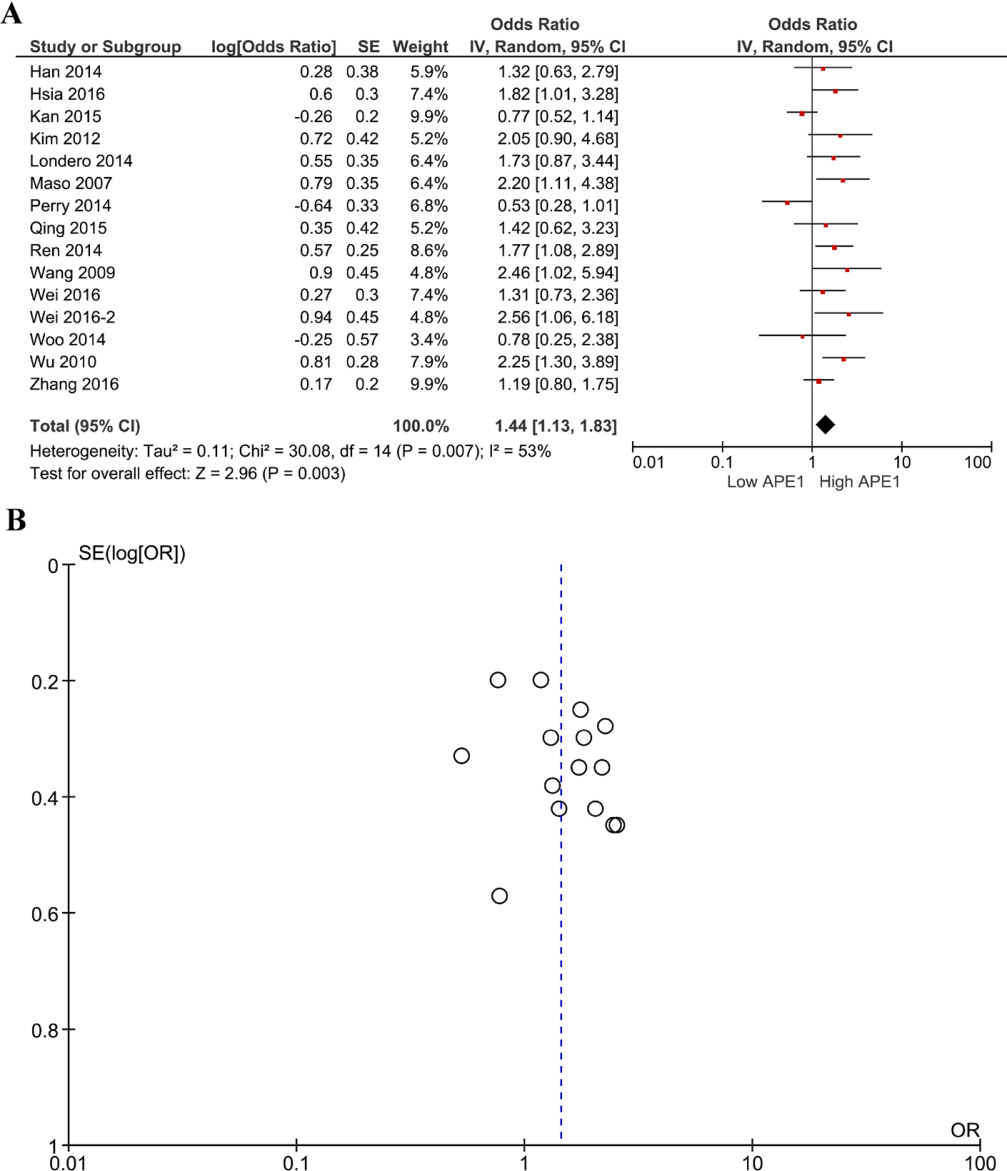
Forest plot (**A**) of the hazard ratio for the association of APE1 levels and OS; Begg's funnel plots of publication bias (**B**) for the meta-analysis of the hazard ratio for the association of APE1 levels and OS.

The correlation between APE1 expression and OS was calculated, and heterogeneity was found among the studies (I^2^ = 53%, *P* = 0.007); therefore, a random model was applied to calculate the pooled HR and its 95% CI. The combined analysis of 15 studies showed that APE1 overexpression was significantly correlated with shorter OS in solid tumors (HR 1.44, 95% CI 1.13–1.83, *P* = 0.003) (Figure 2A).

**Figure 3:**
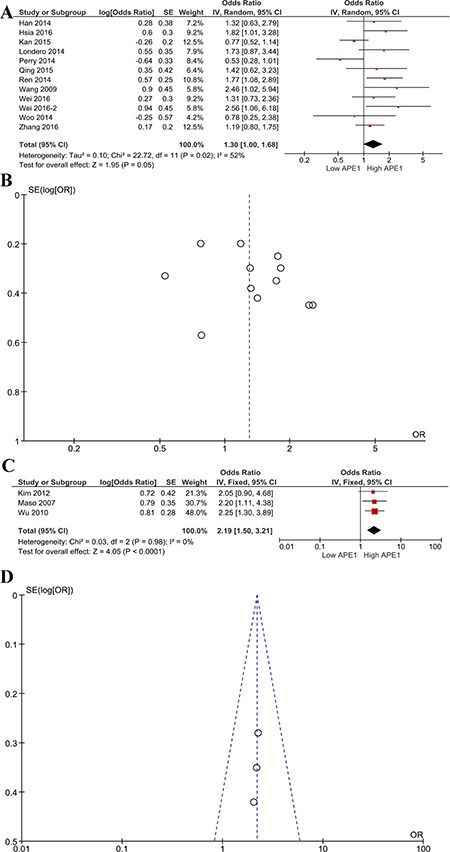
Subgroup analysis of APE1 nuclear/cytoplasmic expression and OS Forest plot (**A**) of the hazard ratio for the association of nuclear-expressed APE1 levels and OS; Begg's funnel plots of publication bias (**B**) for meta-analysis of OS of high and low nuclear-expressed APE1; forest plot (**C**) of the hazard ratio for the association of cytoplasm-expressed APE1 levels and OS; Begg's funnel plots of publication bias (**D**) for meta-analysis of OS of high and low cytoplasm-expressed APE1.

In the subgroup analysis, nuclear-expressed APE1 in 12 studies was correlated with shorter OS (HR 1.30, 95% CI 1.00–1.68, *P* = 0.05, Figure 3A). In the three studies finding expression in only the cytoplasm, APE1 overexpression also showed significant association with shorter OS (HR 2.19, 95% CI 1.50–3.21, *P* < 0.0001, Figure 3C).

**Figure 4:**
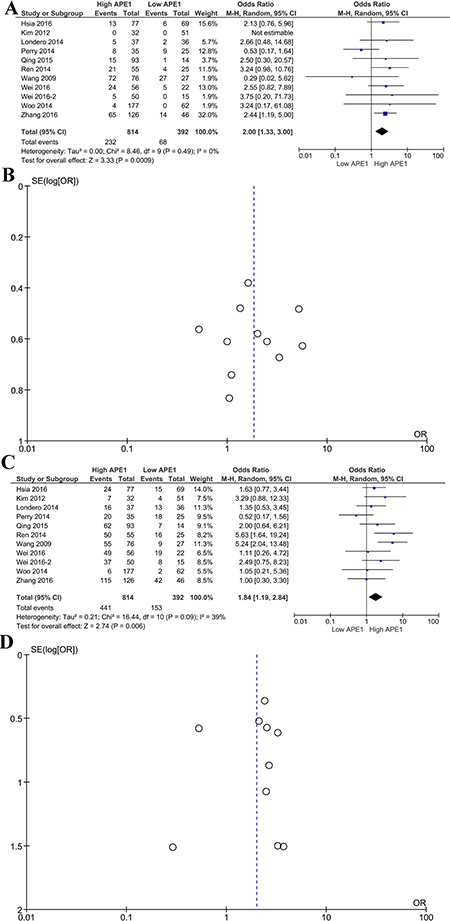
Subgroup analysis of 1-year and 3-year survival rates and APE1 expression Forest plot (**A**) of the hazard ratio for the association of APE1 levels and 1-year survival rate; Begg's funnel plots of publication bias (**B**) for meta-analysis of 1-year survival rate of high and low APE1 expression; forest plot (**C**) of the hazard ratio for the association of APE1 levels and 3-year OS; Begg's funnel plots of publication bias (**D**) for meta-analysis of 3-year survival rates of high and low APE1 expression.

To further analyze the effects of APE1 on survival in tumor patients, the one-year and three-year survival rates were extracted from 10 studies. We investigated the effects of APE1 expression on short and long term survival in tumor patients. Patients with high APE1 expression had lower 12-month survival rates than those with low APE1 levels (HR 2.00 95% CI 1.30–3.00, *P* = 0.0009, Figure 4A). Similarly, overexpression of APE1 was correlated with a lower 36-month survival rate among cancer patients (HR 1.84 95%CI 1.19–2.84, *P* = 0.006, Figure 4C).

### Publication bias

Funnel plots were performed to assess the publication bias in this meta-analysis. Publication bias can be observed in only the one-year survival rate analysis; other major analyses did not reveal obvious evidence of asymmetry (Figures 2B, 3B, 3D, 4B, 4D).

## DISCUSSION

In this study, we meta-analyzed the prognostic role of APE1 in different types of solid tumors. The results show that overexpression of APE1 acted as an unfavorable prognostic factor for solid tumors.

Dysregulation of APE1/Ref-1 has been reported to be associated with cancers for 20 years. Both nuclear and cytoplasmic expression levels of APE1 can be altered in cancers, but it is still unclear how nuclear and cytoplasmic expression of APE1 specifically alter and affect different cancers [22, 23]. Most studies propose that high APE1 is associated with poor prognosis. However, some manuscripts noted that low APE1 was independently associated with poor prognosis [12]. Thus, a meta-analysis of the prognostic role of APE1 is very important. Based on our meta-analysis, regardless of its subcellular location, the expression of APE1 is prognostically significant. To explore the differences in nuclear and cytoplasmic APE1 expression, we continued the subgroup analysis by dividing the included papers into nuclear and cytoplasmic groups and found that both types of APE1 expression are important indicators of poor prognosis. This result not only implies the pleiotropic function of APE1 in mammalian cells but also calls for further research of cytoplasmic APE1. Although its cooperation with AP1, EGR1 and other onco-transcription factors has been discovered, its functions in the cytoplasm remain largely unknown. One explanation of its cytoplasmic accumulation is that Cys93 and Cys310 of APE1 can be S-nitrosated when stimulated by nitric oxide, leading to the cytoplasmic relocalization of the protein [24]. In lung cancer, it is reported that nuclear factor-kappa B is activated by cytoplasmic APE1 [21, 25]. Our meta-analysis results showed more evidence for the clinical significance of cytoplasmic APE1.

We noticed that there were 3 studies demonstrating a better prognosis based on APE1 overexpression in breast cancer, glioma and HCC. However, we found that in the breast cancer paper, there was no significant difference in OS rates between the APE1 high-level expression group and the APE1 low-level expression group (*P* = 0.294). In the HCC paper, the authors suggested that the correlation of APE1 expression with P53 and PD-L1 protein levels might account for the better prognosis, but the *p* value was 0.182, which failed to reach statistical significance. What is more, some recent transcriptional and basic studies of APE1 in hepatocellular carcinoma identified it as an oncogene [26, 27]. Therefore, the better prognosis could be a result of heterogeneity of tumor cells and samples used in the HCC paper. Regarding the glioma report, the authors speculated that APE1 operates in conjunction with other DNA repair molecules, such as NBN, PMS2, MGMT and PTEN, to influence biological and clinical outcomes in glioma. All these findings suggest that more studies of APE1 in these types of tumors are necessary.

There are some limitations of this meta-analysis. First, the number of included studies is small, and some important tumors, including bladder, renal cell and prostate cancers, have yet to be explored. More studies are needed to reveal the role that APE1 plays in these kinds of tumors. Additionally, the cutoff values used to define high and low APE1 levels in the included studies varied, which increased the heterogeneity of our meta-analysis study. ELISA-based serum detection of APE1 levels might be a good approach in the future. Finally, although obvious publication bias was not observed in our meta-analysis, potential bias might still exist since positive results were more likely to be published.

In conclusion, we demonstrated that elevated expression of APE1 detected by IHC is significantly associated with poor survival in numerous types of cancer. APE1 could be a potential biomarker for predicting prognosis in patients with solid tumors.

## MATERIALS AND METHODS

### Literature search

A comprehensive literature search for studies on the prognostic effects of APE1 expression in solid tumor patients was conducted. The keywords used for the search were “cancer”, “carcinoma”, “apurinic/apyrimidinic endonuclease-1”, “APE1”, “HAP1”, “prognosis”, and “survival” (variably combined). The literature searches were performed on Pubmed, Web of Knowledge and Embase platforms The last search was conducted on 6 January 2017.

### Study inclusion/exclusion criteria

All of the included studies in this manuscript met the following requirements: (a) published studies contained the measurement of APE1 expression in the patients with any type of adult carcinoma; (b) overall survival was included as an outcome; (c) APE1 expression was assessed by immunohistochemistry; (d) studies were published within 10 years, from 2007 to 2017; (e) studies reported hazard ratio (HR) estimates with 95% confidence intervals or the HR with 95% CI could be calculated from Kaplan-Meier survival curves; (f) the literature search was limited to studies published in English.

Studies were excluded based on the following criteria: (a) review articles, case reports, duplicated studies, conference abstract or letters; (b) studies providing insufficient survival outcome data; (c) APE1 expression was evaluated using a method other than IHC. The reviewers were blinded to the authors and institutions of the studies undergoing review.

### Data extraction

Two independent researchers, Chun-Ling Yuan and Yan Lin, independently extracted data from all eligible publications according to the criteria shown above. The following information, including the first author's name, year of publication, country of origin, tumor type, number of patients, TNM stage, follow-up period, staining location, prognostic outcomes of interest, analytical method, 12-month survival rates, 36-month survival rates and HR with its 95% CI (if both univariate and multivariate analyses were performed, HRs were extracted from multivariate analyses). Engauge Digitizer 4.1 software was applied to digitize and extract data when the prognosis information was plotted only as Kaplan-Meier curves. For the extraction of 12- and 36-year survival data, some disagreements occurred between Chun-ling Yuan and Yan Lin during the digitization of survival data. We solved these by maximizing the quality of the Kaplan-Meier plots before digitization.

### Quality assessment and statistical analysis

The study's methodology quality was assessed and scored by two independent authors (Fan He and Hui-Ni Wu) according to the Newcastle-Ottawa quality scale (Ottawa Hospital Research Institute). Each study was given a score ranging from 0 to 9 after discussion between the two authors.

The impact of APE1 overexpression on patient survival in solid tumors was estimated by the HR and its 95% CI and one- and three-year survival rates. The one- and three-year survival rates of each study were extracted from Kaplan-Meier curves. Statistical heterogeneity between studies was quantified using Cochran's Q test and Higgins I-squared statistic. Heterogeneity was defined as *P* < 0.1 or I^2^ > 50%. In the absence of statistically significant heterogeneity, a fixed effects model was selected to combine the data. Otherwise, a random-effects model was used. Publication bias was assessed by using Begg's funnel plot test. For all analyses, a two-sided *P* value less than 0.05 was considered statistically significant. All analyses were performed with Cochrane Review Manager version 5.3 (Cochrane Library).
